# Is Dynamic Autocrine Insulin Signaling Possible? A Mathematical Model Predicts Picomolar Concentrations of Extracellular Monomeric Insulin within Human Pancreatic Islets

**DOI:** 10.1371/journal.pone.0064860

**Published:** 2013-06-14

**Authors:** Minghu Wang, Jiaxu Li, Gareth E. Lim, James D. Johnson

**Affiliations:** 1 Department of Mathematics, University of Louisville, Louisville, Kentucky, United States of America; 2 Department of Cellular and Physiological Sciences and Department of Surgery, University of British Columbia, Vancouver, BC, Canada; University of Bremen, Germany

## Abstract

Insulin signaling is essential for 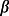-cell survival and proliferation *in vivo*. Insulin also has potent mitogenic and anti-apoptotic actions on cultured 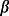-cells, with maximum effect in the high picomolar range and diminishing effect at high nanomolar doses. In order to understand whether these effects of insulin are constitutive or can be subjected to physiological modulation, it is essential to estimate the extracellular concentration of monomeric insulin within an intact islet. Unfortunately, the *in vivo* concentration of insulin monomers within the islet cannot be measured directly with current technology. Here, we present the first mathematical model designed to estimate the levels of monomeric insulin within the islet extracellular space. Insulin is released as insoluble crystals that exhibit a delayed dissociation into hexamers, dimers, and eventually monomers, which only then can act as signaling ligands. The rates at which different forms of insulin dissolve *in vivo* have been estimated from studies of peripheral insulin injection sites. We used this and other information to formulate a mathematical model to estimate the local insulin concentration within a single islet as a function of glucose. Model parameters were estimated from existing literature. Components of the model were validated using experimental data, if available. Model analysis predicted that the majority of monomeric insulin in the islet is that which has been returned from the periphery, and the concentration of intra-islet monomeric insulin varies from 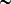
50–300 pM when glucose is in the physiological range. Thus, our results suggest that the local concentration of monomeric insulin within the islet is in the picomolar ‘sweet spot’ range of insulin doses that activate the insulin receptor and have the most potent effects on 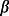-cells *in vitro*. Together with experimental data, these estimations support the concept that autocrine/paracrine insulin signalling within the islet is dynamic, rather than constitutive and saturated.

## Introduction

There is increasing evidence that insulin has critical autocrine or paracrine feedback actions within pancreatic islets [1], [2]. There are also data that suggests that human islets may undergo insulin resistance in type 2 diabetes [3], potentially unifying the etiology of type 2 diabetes. Two lines of evidence have been presented that suggest that 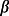-cell insulin signaling is physiologically relevant. First, *in vivo* loss-of-function studies demonstrate that 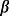-cell insulin signaling plays an important role in the control of 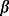-cell apoptosis, proliferation and mass. For example, Kulkarni's group has shown that knockout of the 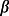-cell insulin receptor reduced functional 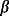-cell mass and increased apoptosis [4],[5],[6],[7]. Moreover, the same group has demonstrated that 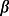-cell proliferation in response to both genetic and diet-induced insulin resistance was absent in mice lacking 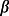-cell insulin receptors [8]. Our observation that physiological hyperinsulinemia is required for the high fat diet-induced increase in 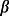-cells implies sensitivity to dynamic changes in insulin [9]. Complementing these *in vivo* loss-of-function studies, we and others have shown that exogenous insulin protects primary human and mouse islet cells from serum-withdrawal-induced apoptosis, via NAADP-dependent Ca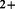 release, a signalling hub involving 14-3-3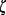, Raf1, Mek, Erk, and Bad, as well as master transcription factors such as Pdx1 and Foxo1 [10],[11],[12],[13],[14]. We also showed that insulin, but not glucose, directly increases proliferation of primary mouse dispersed islet 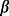-cells [15]. These observations complement the work of multiple groups that suggest that insulin promotes 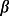-cell survival. Whether insulin can affect 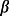-cell secretory function or protein synthesis acutely is more controversial [16] and may depend on the dose, duration and context of the insulin signal [17].

Perhaps the most unexpected observation of our previous studies was that the dose-response relationships between insulin and apoptosis or proliferation did not follow a typical dose-response relationship [1]. We have consistently observed that lower doses of insulin, typically 200 pM, were more effective than higher doses in the nanomolar range [10],[11],[12],[13],[18]. However, the *in vivo* relevance of signaling events activated by picomolar insulin doses has been questioned due to the speculative assumption that 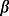-cells are exposed to extremely high levels of insulin within the islet. Although it may seem intuitive to some that local insulin levels would be high near the 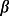-cell, there is no direct evidence for this. The insulin concentration in the native 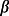-cell microenvironment has never been measured experimentally. The physically closest insulin measurements to the islet *in vivo* are those of the portal vein (400–1200 pM depending on the glucose concentration) [19], but this represents the net insulin release from 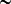
1 million islets dispersed throughout the pancreatic parenchyma (40 cm^3^) [20] concentrated into the relatively small volume of a single vein. Moreover, there are two important clues that support the possibility that 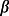-cells may be exposed to insulin concentrations less than that of the portal circulation. First, some 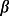-cells must be exposed to circulating blood first, before other cells, within the complex microvasculature of the islet [10],[21], and would therefore be exposed to insulin levels identical to non-endothelial cells bathed by the peripheral circulating insulin, which is 40–100 pM at rest and 400 pM after a meal [19]. Second, it is known that insulin is stored and secreted as an insoluble microcrystal, dissolving only when exposed to the pH of blood [22]. This means that the amount of local monomeric insulin capable of binding to 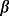-cell receptors depends on both the secretion rate and the rate at which insulin crystals dissolve into active monomers. There is also the argument that 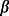-cells would be optimally tuned to the range of insulin they see *in vivo*, and we have shown they are most responsive at 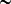
200 pM [10],[11],[12],[13],[18]. Insulin binding assays have shown that the IC50 of the ligand for the insulin receptor is in the picomolar range (88 pM) [23]. Given the current hurdles to experimentally measure the intra-islet insulin concentration, a powerful alternate approach is to undertake quantitative estimates using mathematical modeling [24]. We expect that the technology to measure insulin monomer levels *in vivo* will eventually be developed and permit our predictions to be tested experimentally.

An accurate model to distinguish the contribution of peripheral insulin and newly secreted insulin on 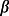-cell dynamics requires detailed knowledge of a number of key parameters, including the rate of insulin secretion and the rate by which insulin crystals convert to monomers. In this work, local insulin refers to the newly synthesized and released insulin while peripheral insulin refers to insulin in circulating blood re-entering islets. The insulin surrounding the 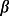-cell is therefore a blend of these two concentrations. After synthesis, insulin is packaged in dense-core secretory granules as an insoluble zinc-associated crystal and is released following Ca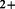-dependent exocytosis in response to glucose [22],[25]. At high concentrations, such as those contained in therapeutic insulin formulations, insulin is primarily aggregated into hexamers as the default state [26]. Two sequential steps are required for the dissolution of the released insulin crystal into monomers, which interact with their cognate receptors. Each insulin hexamer must first dissolve into three dimers, followed by the breakdown into six insulin monomers [26],[27],[28]. With these delays, a fraction of non-monomeric insulin may flow and/or diffuse away from the immediate vicinity of the 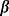-cells. Therefore, the concentration of the local monomeric insulin that contributes to active signaling could be lower than the total insulin released. Mathematical modeling is therefore an important in silico tool for biologists to better understand the properties and actions of insulin *in vivo* in sites where it cannot be measured directly. Here, we report the outputs from the first such model, which illustrates the possible kinetics of the endogenous local insulin near 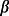-cells. This work provides new insight into whether signaling events activated by high picomolar insulin might be relevant *in vivo*.

## Results

### Model formulation and logical considerations

The islets of Langerhans are clusters of 50–1000 endocrine cells. They are dispersed throughout the pancreas and represent 1–2% of the total pancreatic volume. Pancreatic islets are highly vascularized micro-organs, with a central core of 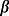-cells surrounded by other endocrine cells, although this organization is less strict in adult human islets [21] or 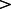
1 year-old adult mouse islets [29]. Insulin secreting 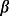-cells comprise the majority (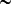
50–70%) of islet endocrine cell types, although this can depend on the islet and on the individual [30]. A pancreatic islet is composed of 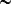
65% endocrine cells, and 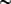
35% extracellular space/vasculature [31].

**Figure 1:**
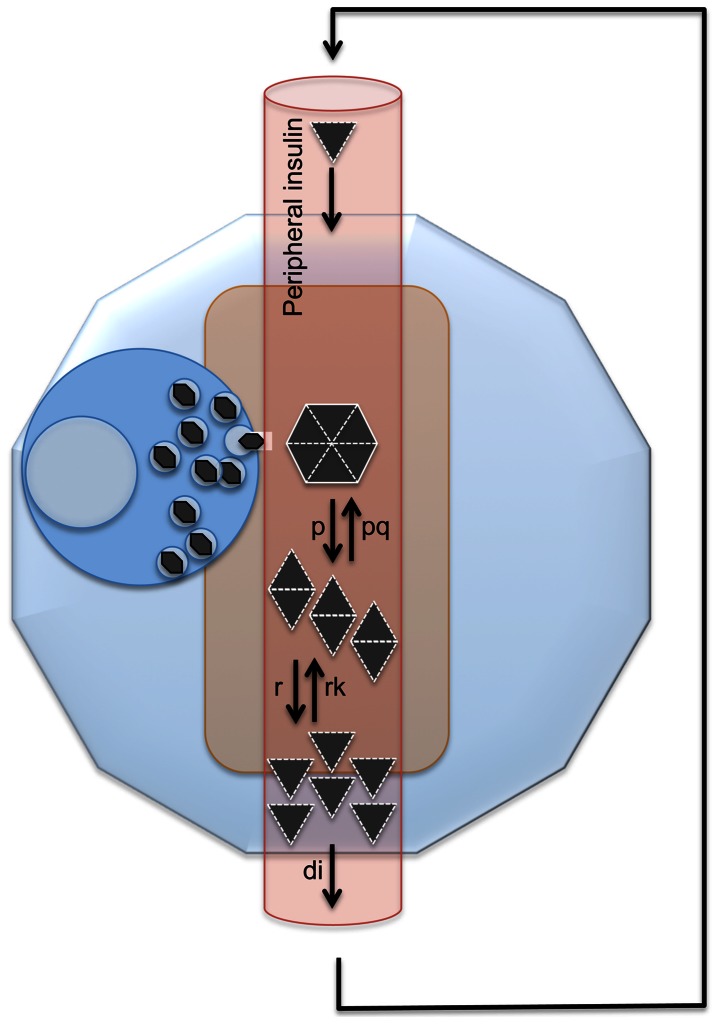
Model diagram for estimation of islet insulin concentration of hexamers, dimers, and monomers. This diagram reflects that, in an intact pancreatic islet, newly synthesized and released hexameric insulin dissolves into dimers and then monomers. Newly released insulin moves out of pancreas into peripheral circulation and then returns the islet with blood flow as ‘old’ peripheral insulin.

**Figure 3:**
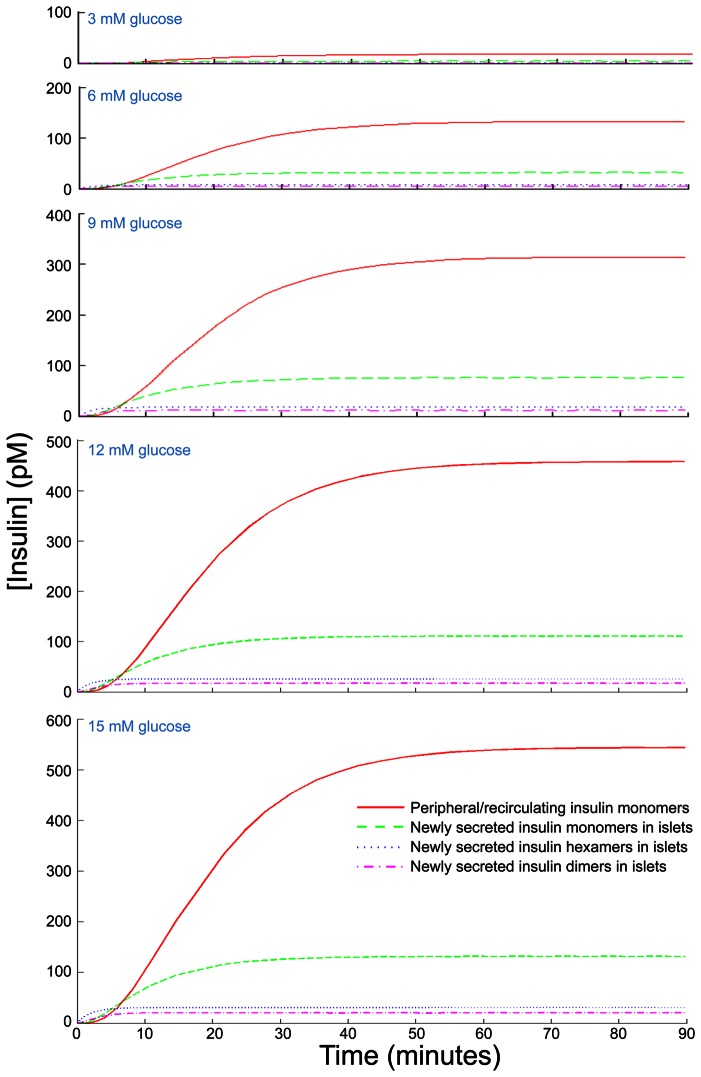
Numerical simulations of insulin hexamer, dimer and monomer concentration kinetics in a model human islet at multiple glucose concentrations. Profiles show the dynamics of the concentrations of newly synthesized insulin (in hexamer 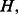 dimer 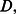 monomer 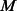) and returned peripheral insulin 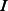 and the equilibrium (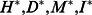) (pM) at different clamped glucose inputs 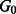 (mM).

**Figure 4:**
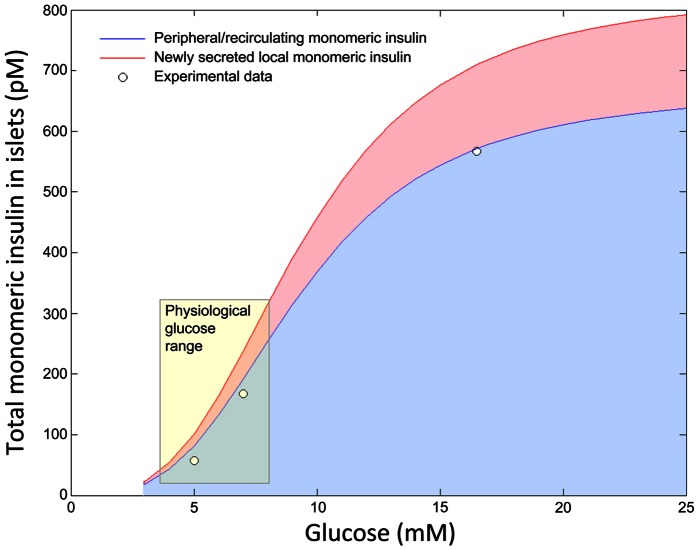
Total insulin monomer levels in a model islet resulting from the combination of new insulin and returning insulin. The dose-response relationship between glucose and total islet monomeric insulin. Newly released monomeric insulin (pink) plus returning peripheral monomeric insulin (blue) illustrates the total levels of monomeric insulin within islets. The white dots represent validation data from Polonsky et al 1988 ([43]).

The present model is built on the logical assumption that the kinetics by which insulin crystals dissolve plays a critical role in the local islet insulin concentration. The release of intact insulin crystals from single 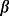-cells has been demonstrated by multiple groups (e.g. Fig. 3I, in reference [22]), and it follows that it must take some time for these crystals to dissipate after exocytosis (Fig. 4F in reference [22]). The rate at which insulin hexamers dissolve into dimers and then monomers after subcutaneous injection at a point source has previously been addressed by our group and others (Fig. 1 in Li and Kuang [27]) and it happens on a time-scale of hours [26], [32]. The *in vivo* half-life of insulin is known to be 5–6 minutes [33], which means virtually all of the body's insulin must be replaced by the 1 million islets in 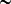
10 minutes. The baseline peripheral insulin concentration in the whole body circulation is 50–80 pM. While we are not aware of studies that have directly measured insulin levels in arteries flowing into the pancreas, one would assume that non-degraded peripheral insulin returns to the pancreas at concentrations similar to any other organ. In addition to the prominent insulin degradation in the periphery, we also expect significant insulin re-uptake and degradation in islets, since 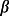-cells are highly enriched with insulin receptors [12] and the endocytosis of insulin: insulin receptor complexes leads to their degradation in lysosomes (T. Albrecht, J.D. Johnson, unpublished data).

We initially considered whether the rate of blood flow in the pancreas might also be an important component of the model. Arterial blood flows into the pancreas through an artery connected to the aorta and flows out of the pancreas via the portal vein after passing through a network of fine capillaries. Although it was originally though that islet blood flow always proceeded from the inside out, recent *in vivo* imaging of intra-islet blood flow in mice has revealed multiple patterns of local islet blood flow (Fig. 5 in Meier et al. [21],[34]). There is also evidence that blood can fill some whole islets evenly without a flow direction (Fig. 4 of Nyman et al. [21]). Regardless of the direction, each case will have some ‘prime’ or ‘upstream’ 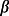-cells exposed to fresh arterial blood with other 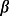-cells subsequently exposed to locally released insulin. The percentage of 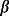-cells exposed to fresh arterial blood is related to the width of an islet, which is relatively uniform and conserved throughout evolution. For simplicity, we will assume that, on average, the blood flow in the islet is irregular relative to the location of a given 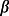-cell. Islet capillaries have been shown by electron microscopy to possess very thin endothelial cells, which are highly fenestrated and permeable [35], suggesting that the local barriers to the movement of insulin crystals, hexamers or dimers are negligible. For the sake of simplicity, this initial model will therefore not consider physical barriers between the 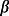-cell and the circulation. Newly released insulin flows out of the pancreas and into the liver through the hepatic portal vein within a few seconds. The mean portal vein blood flow rate has been measured between 0.8 and 1.1 L/min, and no changes are observed between the basal state and a hyperglycemic clamp [19]. The total rate of pancreatic blood flow through the entire pancreas has been estimated using computed tomography to be 1.5 ml/min per ml of pancreas tissue [36]. Since we only consider a single prototypical islet as the point source in this model, and blood flow does not need to be considered, as its rate is relatively constant in and out of the islet. In other words, the dilution of ‘islet blood’ with re-circulating insulin in peripheral blood cancels out the addition of new insulin to the blood downstream of the islet.

**Figure 5:**
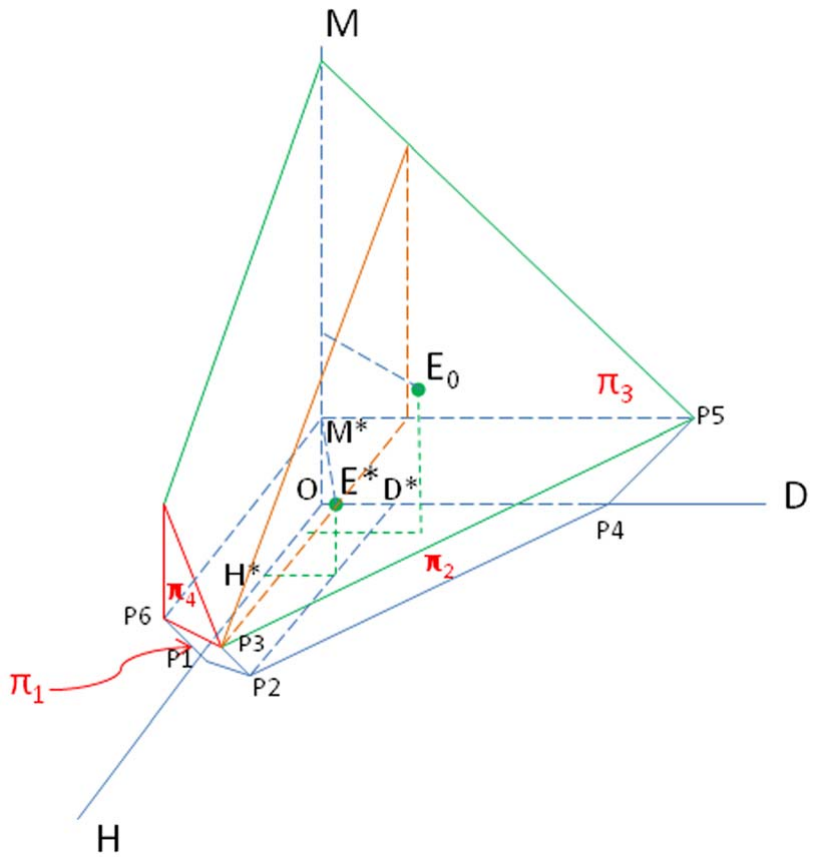
The bounded region 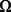 for the proof of Theorem 1.

All of the above considerations are summarized in the single islet model diagram (Fig. 1). These can also be summarized in the following word equation system for this model that considers a single islet and follows the law of mass action and the law of conservation. The rate of change of the concentration of insulin in any form can be expressed as the
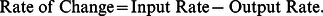



We denote the concentration of insulin hexamers by 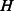 (in pM), dimers by 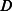 (in pM), monomers by 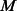 (in pM), and peripheral insulin monomers returned to the islet by 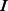 (in pM). The transfer of peripheral insulin monomers should be same as the monomeric insulin concentration that remains in the circulation. We therefore obtain the following model for relative concentrations of local free insulin hexamers, dimers and monomers, and peripheral insulin:
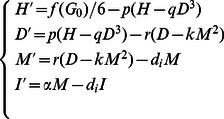
(1)with the initial condition 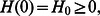 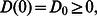 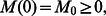 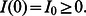 The transport from newly secreted insulin to peripheral insulin is assumed to be proportional to the level of monomers, 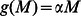, 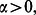 and the secretion of insulin hexamers is stimulated by glucose, which follows the dynamics of Hill's function 
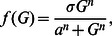
 which has been employed in other models [27], [37], [38], [39], [40], [41]. The division of 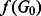 by 6 is due to the secretion of insulin from the 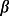-cells as insulin hexamers. All parameters have the following meanings.


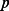
 (min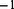) – the dissolution rate from hexamer to dimer.


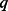
 (L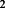/pmol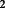) – the coefficient of the aggregation from dimer to hexamer.


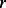
 (min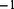) – the dissolution rate from dimer to monomer. It is faster for a dimer to dissolve into two monomers than for a hexamer to dissolve into three dimers. Hence 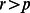.


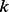
 (L/pmol) – the coefficient of the aggregation from monomer to dimer.


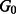
 (mM) – the clamped glucose concentration (normal *in vivo* range is 3.9–6.1 mM).


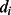
 (min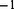) – the degradation rate of insulin (both local free and peripheral).


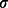
 (pM/min/mM) – maximum secretion rate of insulin stimulated by glucose.


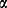
 (min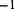) – the transfer rate of newly secreted insulin to peripheral insulin.


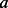
 (mM) – half-saturation point in Hill's function 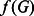.

### Model analysis

Next, we studied the qualitative behaviors of the model (1). The model has a unique equilibrium 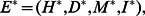 given by
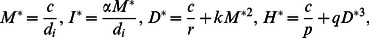
(2)where 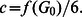 Since the system (1) is cooperative, it is intuitive that the unique equilibrium is globally stable. To this end, we first obtain the following:

**Theorem 1** *Any solution of the model (1) with initial condition, 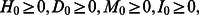 is positive and bounded.*

The proof of this analytical result can be found below. Then, with direct application of Theorem 1, and Theorem 2.3.1 (in Smith, HL, page 8 of reference [42]), we obtain the following theorem that qualitatively ensures the reliability and robustness of our model. In next section we further demonstrate that our numerical analysis is quantitatively reasonable.

**Theorem 2** *The unique equilibrium point 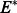 is globally asymptotically stable.*

It can be seen by (2) that, at equilibrium, the concentrations of local insulin 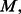 peripheral insulin 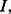 hexamers and dimers depend on 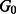 in nonlinear relationships. However, the ratio of the newly synthesized insulin and the peripheral insulin at equilibrium can be expressed by
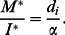
(3)


The equation (3) indicates that the ratio of the concentration of local free insulin and peripheral insulin is linearly dependent on insulin degradation 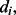 and inversely proportional to the transfer rate 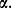 On the other hand, insulin concentration of intra-islet can be estimated by
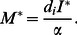
(4)


### Parameter estimation and numerical simulations

As detailed in the following section, model parameters were selected based on the existing literature, regression fitting of experimental data found in the literature, or reasonable assumptions from known physiological principles. Using the resulting model, we then simulated insulin secretion dynamics under conditions of various clamped glucose inputs. We chose the initial condition of the model as 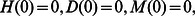 and 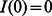 as Theorem 2 guarantees that the equilibrium is globally stable and thus the initial conditions do not affect the equilibrium.

**Figure 2:**
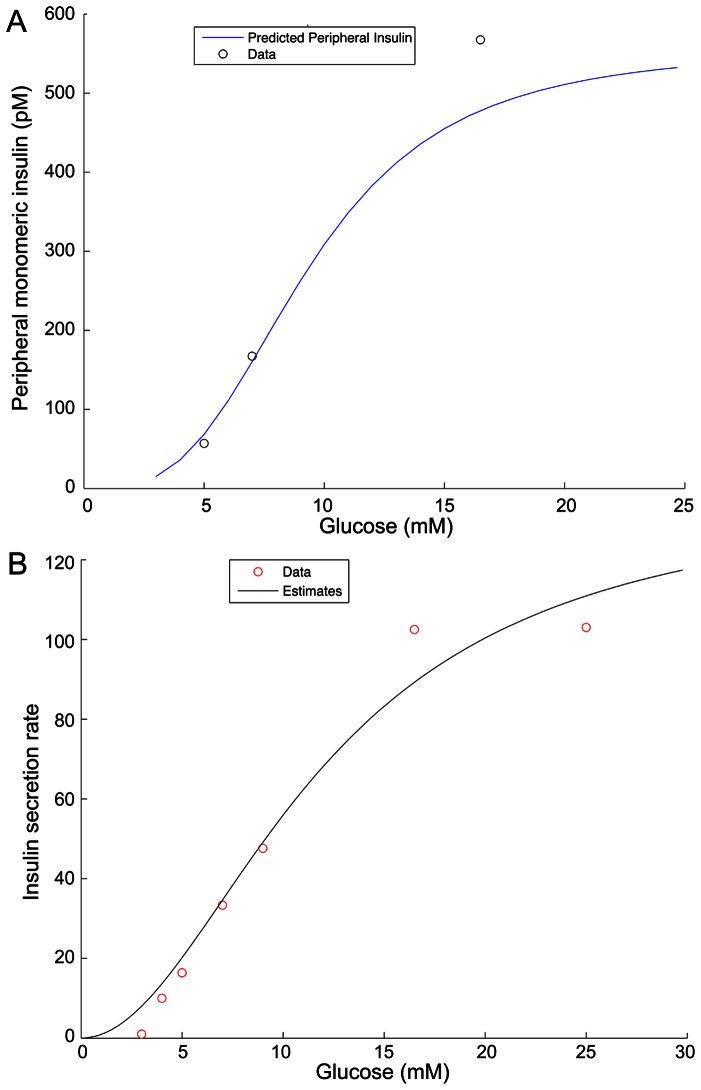
Validation of the model insulin secretion rate function. (**A**) The model output plotted against dose response data from Polonsky et al (1988) for comparison. (**B**) Insulin secretion rate data were use to estimate an insulin secretion rate response function. Model profiles were then created with different doses of clamped glucose (mM) and compared to peripheral insulin values (pM) at equilibrium at those glucose doses (mM).

The estimation of insulin secretion rate 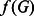 was as follows. It is widely accepted that the insulin secretion response to glucose takes a sigmoid shape function, namely Hill's function 
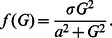
 To estimate the parameters 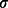, 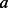 and 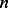, we primarily used published data of *in vivo* insulin secretion rates at various glucose levels in human subjects [43], [44] as shown in following Table 1 by employing the Least Square Method. Additional points on the curve were estimated and added for fitting. The fitting results in 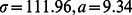 and 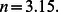 Panel (B) in Fig. 2 illustrates the fitted curve. It is noteworthy that the estimated values are in agreement to those estimated in IVGTT models ([37], [38], [39]). As mentioned earlier, this secretion rate should be divided by 6 to obtain the secreted concentration of insulin hexamers.

**Table 1:** Insulin secretion data rate and multiple glucose concentrations.

Glucose level (mM)	Secretion rate (pM/min)	References and Notes
3	1	Data point estimated and added for fitting
4	10	Data point estimated and added for fitting
5	16	The case of overnight fast in [43]. Glucose level ranges between 4 and 6 mM after an overnight fast. We chose 5 mM.
7	33	The case of 24-h on mixed diet in [43]. We assume at 7 mM.
9	47	[44]
16.5	102	The case of hyperglycemic clamp at 16.5 mM in [43].
25	103	Data point estimated and added for fitting

The estimation of 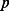 and 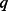 was as follows. We estimated the values of the parameters 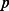 and 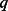 for human insulin based on data from insulin analogues, with the assumption that the dissolution and aggregation rates of human insulin falls between the rates associated with fast insulin analogues and slow insulin analogues. According to Li and Kuang, 2009 [27]; Tarin et al 2005 [45] and Trajanoski et al 1993 [32], the value of the parameter p (min^−1^) remains the same for fast and slow insulin analogues. So we assume that 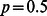 (min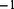) for human insulin as well. Table 2, adopted from Tarin et al 2005 [45], shows the values of the parameter 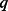 (L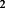/pM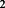) for various insulin analogues. Therefore, the parameter 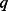 for human insulin is estimated as the value for Semilente, i.e., 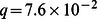 ml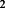/U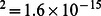 L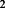/pmol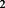 after unit conversion by a factor 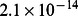.

**Table 2:** Values of parameter q for different insulin formulations.^1^

Units	Lispro	Actrapid	Semilente	NPH, Glargine
ml^2^/U^2^	4.8×10^−4^	1.9×10^−3^	7.6×10^−2^	3.0
L^2^/pmol^2^	9.8×10^−18^	3.9×10^−17^	1.6×10^−15^	6.3×10^−14^

1 This table is adopted from Table IV in Tarin et al [45].

The estimation of 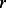 and 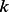 was as follows. It is intuitive that it is faster for a dimer to dissolve to two monomers than for a hexamer to dissolve to three dimers. So it is reasonable to assume 
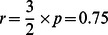
 min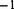 and 
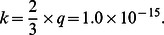


The estimation of 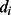 was as follows. Values for the degradation rate 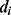 of insulin have been reported within a wide range between 0.03–0.2 ([46]). To select a reasonable value for 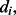 observing that in general a substrate decays exponentially, we considered the following ordinary differential equation,
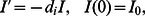
where the initial condition represents the case of no secretion. The solution of this differential equation 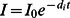 represents the change of amount of insulin over time. Notice that the half life of insulin is approximately in a range of 4–6 minutes [33]. Assuming that the half-life of insulin is 6 min, when 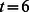 min, 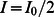 and thus 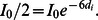 Solving this equation for 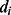 yields 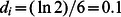 min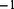.

The estimation of 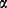 was as follows. We estimated the parameter 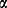 (fraction coefficient of peripheral insulin) as follows. As expressed in equation (1), the rate of change of the returned peripheral insulin concentration in an islet, 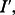 is affected by its degradation 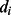 and the concentration of newly secreted insulin monomers, 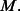 Assume that 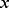 units of insulin monomers are secreted by a single islet. Thus, a total of 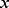 million units of insulin will be released into the portal vein from the one million islets of the pancreas, after which it becomes peripheral insulin. It is established that 50% of secreted insulin is removed from the circulation by the liver on the first pass. We assume an insulin degradation rate of 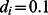 min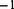 according to the estimation of 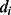 given above. Since it takes about 25 seconds for a complete circulation of blood, newly released insulin must return to the pancreas in the same time, where it is assumed to be evenly distributed among the one million islets. So, approximately 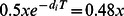 units of insulin return to the same islet. Therefore, we estimate that 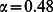 min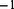.

Using our model with the above carefully selected parameter values, we simulated the dynamics of the various insulin concentrations at a range of clamped glucose concentrations. A stepwise increase in glucose from 0 mM to any value we tested resulted in an increase in peripheral insulin that reached a maximum in 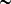
30 minutes (Fig. 3). As expected, the aggregated forms reached their peak faster. The concentration of monomeric insulin that has been newly release reaches only 100 pM after a jump to 15 mM glucose (a glucose concentration is much higher than what is seen in humans after a meal).

Importantly, we are able to use our model to estimate the total concentration of insulin monomers within the islet (i.e. newly made insulin + insulin returning from the periphery). By (3) and the parameter values we estimated above, we can derive the total monomeric insulin present in the islet at any glucose concentration. Simple computation shows that 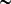
75% of local monomeric insulin is peripheral insulin while 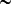
25% is new monomeric insulin (Fig. 4). Our simulations clearly show that the majority of insulin in an islet is returned as monomeric peripheral insulin, whereas the newly secreted monomeric insulin from the islet provides a minor contribution in all cases tested. Therefore, our model predicts that the levels of insulin monomers in the islet do not exceed the picomolar range, even in extreme clamp studies (Fig. 4). Physiological meal-induced glucose excursions rarely fall outside the 4 mM to 8 mM range in normal humans, and our model suggests that monomeric insulin is unlikely to exceed 300 pM in these conditions (Fig. 4).

## Model Validation

We validated the glucose-response component of our model by comparing the model-predicted values of glucose-stimulated insulin responses with the experimental data in [43]. The comparison is shown in Table 3 and Panel (A) in Fig. 2. It is well known that a swift increase of secretion occurs when glucose concentrations rise higher than fasting levels, but becomes saturated beyond the hyperglycemic range. No model can predict true physiology perfectly, but we suggest that this verification is valid because the response function of insulin secretion rate is obtained statistically according to the experimental data. The model-predicted dose response is in approximate agreement with the observed dose response in the same experiment. Some aspects of the model simulations, namely the exact concentration of monomeric insulin in the immediate vicinity of intact human islets *in vivo* must await the technology to measure this directly.

**Table 3:** Comparison of predicted dose-response and observed dose-response values.^1^

Glucose level (mM)	Predicted Peripheral Insulin (pM)	Observed Peripheral Insulin (pM)
5	77.7	57.0
7	186.3	167.4
16.5	571.5	567.4

1 Data are from Polonsky et al 1988 [43].

### Proof of Theorem 1

Notice that the first three equations of model (1) do not involve 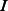, hence we first consider the sub-model
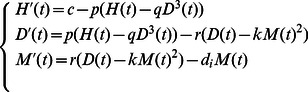
(5)and then the original model (1).

The first quadrant of phase space is invariant. In fact, on the coordinate plane 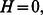 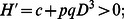 on 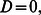 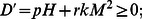 on 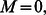 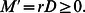 Therefore, trajectories starting from the first quadrant will not cross any of the coordinate plane to negative side.We next construct a bounded region 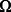 (see Fig. 5) in the first quadrant such that the trajectory 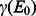 starting from 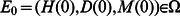 will not cross the boundaries of 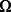; that is, 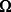 is invariant, thus 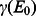 is bounded.We choose 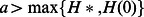 to be large. Let 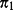 be the plane through the point 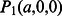 with the normal vector 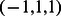. Plane 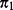 intersects the plane 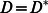 at the line 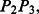 where 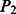 is on the plane 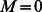 and 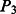 is on the plane 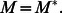 Let 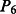 be the intersection point of the plane 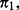 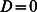 and 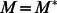. Thus 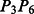 is parallel to 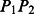, whose direction vector is 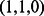. Let 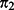 be the plane passing through the line 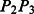 with the normal vector 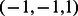. Then 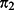 intersects the plane 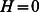 at the line 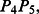 where 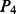 is on the plane 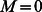, 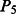 is on the plane 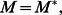 and 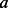 is large enough so that the point 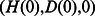 is on the same side of the line 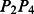 as the origin in the plane 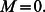 Clearly the line 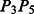 is in the plane 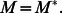 Let 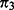 be the plane passing through the line 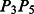 with the normal vector 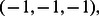 where we require that a was chosen large enough so that the point 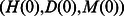 is on the same side of the plane 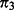 as the origin. Let 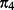 be the plane through the line 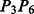 and perpendicular to the plane 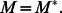 Since 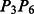 is parallel to 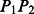 and the direction vector of 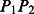 is 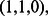 the inward normal vector of 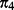 is 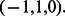 Therefore, the planes 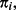 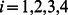 and the coordinate planes 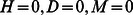 form a closed region 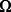 (refer to Fig. 5), and 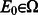.

Now we shall show that the bounded region 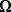 is invariant and thus 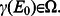 To this end, we need only to show that the inner products of the inward normal vectors of the plane 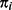, 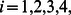 with the direction vector of trajectory starting from these planes are nonnegative.

On 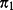, 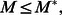 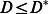 and 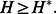


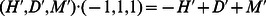


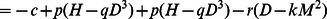


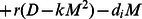


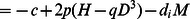


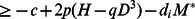


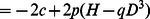


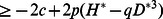


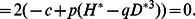



On 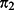, 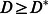 and 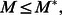 thus we have
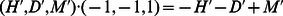


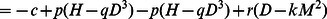


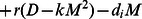


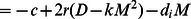


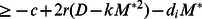


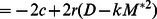


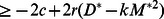


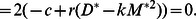



On 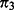, 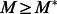, thus we have
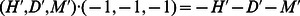


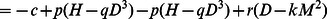


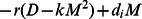


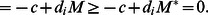



On 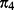, 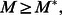 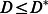 and 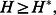 thus we have
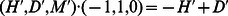


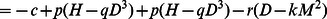


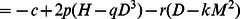


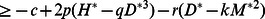


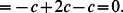



Therefore these inner products are nonnegative, which implies the trajectory on boundaries 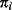, 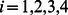 goes inward of 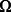. Moreover, we have proved that the first quadrant is invariant (Theorem 1). Therefore, we conclude that 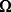 is invariant. It is equivalent to state that the solution to the sub-model 5 is bounded. Now for the model (1), since 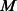 is bounded, thus clearly 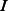 is bounded. We complete the proof.

## Discussion

The goal of the present work was to estimate the local insulin concentrations within a working human pancreatic islet. This estimation is essential for efforts to understand the physiological relevance and mode of islet paracrine insulin signaling. More specifically, it has the potential to elucidate whether signaling is constitutive as a result of saturating local insulin levels or dynamic with monomeric insulin varying in a range that permits optimal activation of the insulin receptor. To the best of our knowledge, this is the first time any such estimation has been systematically generated. Our model suggests that locally produced insulin at steady state makes a relatively minor contribution to the local levels of monomeric insulin in an individual islet (estimated to be 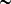
25%). In contrast, the majority of locally available monomeric insulin within the islet derives from newly returned insulin from the peripheral circulation. Regardless, our model indicates that within an islet, the percentage of new insulin converted to monomers before being swept away by the blood flow is always less than the insulin returned from the peripheral circulation. Since the peripheral insulin concentration is well known, we can be confident that the total monomeric insulin concentration in the islet is not in orders of magnitude higher than it is in the periphery, as has been implied by some commentators. The model is dynamic, and our simulations show that the majority of the changes take place within 30 minutes, regardless of the glucose step. Since the initial conditions used in the simulations are somewhat arbitrary, one should not imply too much from the shape of these curves. Nevertheless, it is clear that the monomeric insulin concentration in the islet is relatively low in response to an increase in glucose at each time point before equilibrium is reached. It should also be noted that our model focuses primarily on glucose as the main stimulus for insulin release, and does not exclude modulatory contributions of other nutrients, incretin hormones or neural inputs, which increase insulin levels.

Many studies have investigated the effects of insulin on pancreatic 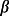-cells; however, the vast majority have employed insulin doses in the high nanomolar range. This, together with culture conditions that allow insulin to accumulate, may be one of the reasons that some investigators have failed to observe significant effects of insulin in their *in vitro* experiments. Our model suggests that such high doses are non-physiological and up to 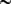
1000 times higher than the locally present levels. Importantly, nanomolar doses of insulin would maximally stimulate IGF1 receptors [47], which do not play an essential role in islet survival and have only a minor role on 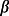-cell function [5],[48]. Our model also suggests that the concentration of free monomeric insulin in the islet may not exceed 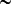
300 pM while glucose is in the physiological range. Thus, local autocrine/paracrine insulin signaling is likely to be dynamic and physiologically relevant in the picomolar range. This also means the experimentally defined ‘sweet spot’ where 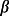-cells respond *in vitro* optimally to picomolar insulin is likely to be well within the *in vivo* physiological range [1]. This might seem counterintuitive, but our model suggests that the slow kinetics by which insulin crystals dissolve keeps the local monomeric insulin levels surprisingly low. Although we have not considered the rate of islet blood flow in our model, it may nonetheless be important and should be considered in future models. Changes in islet vasculature and/or blood flow might therefore represent physiological and pathological modes of altering local insulin signaling.

One important assumption of our model is that the percentage of insulin released in its crystalized form is high. This generally appears to be well supported by the literature, but there are cases where it might not be as assumed here. Some species of rodents (e.g. porcupines) have insulin genes with alterations in amino acids known to be important in zinc binding and crystal formation [49]. In these examples, we might therefore expect an increased ratio of monomeric to hexameric local insulin and possibly a desensitization of islet insulin signaling, but this should be investigated in future modeling studies. Similarly, a human mutation (AspB10) has been described that alters insulin binding to zinc and complex assembly, and we would predict that autocrine insulin signaling might be altered in such patients, in addition to multiple other defects [50]. It would also be interesting to determine if modifications in insulin packaging caused by recently described genetic polymorphisms in zinc transporters might affect the ratio of insulin released in a post-crystalized form under specific conditions [51]. In each of these cases, we expect insulin signaling pathways to compensate in the face of altered monomeric insulin levels [52].

We speculate that our findings also have implications for efforts to understand the evolution and function of the endocrine pancreas. Remarkably, the size of islets is relatively similar between mammals of different body sizes, with the total number of islets varying between small and large species. The islets of Langerhans are also somewhat unique as an endocrine organ in that they are spread throughout the large pancreas in a diffuse, but non-random manner. We speculate that this architecture evolved to prevent the accumulation of high levels of insulin in and around the islets. Insulin is a powerful mitogen [53] and we have shown it can stimulate primary islet cell proliferation [15]. We have recently shown that pancreatic insulin hypersecretion is required for the increase in islet size observed during high fat feeding [9]. Thus, the diffuse distribution of relative small islets might be evolutionarily conserved to prevent excessive local insulin concentrations that might increase the risk of pancreatic cancer. Indeed, significant molecular, clinical and epidemiological findings point to important links between hyperinsulinemia and pancreatic cancer [54], [55], [56], [57], [58].

This work is the first to model the intra-islet concentration of monomeric islet insulin. We conclude that the major contributor to the human islet insulin levels is peripheral insulin owing to the relatively slow dissolution of newly secreted insulin crystals. We find that the total concentration of free monomeric insulin, capable of activating the insulin receptor, remains in the picomolar range regardless of glucose stimulation. Together, these findings add novel insight into pancreatic islet biology and provide context to the experimental studies focused on the effects of islet insulin signaling. Our work helps contextualize *in vivo* and *in vitro* studies on the autocrine effects of insulin and lay the groundwork for future mathematical and physiological studies.
